# Cellular and Humoral Mechanisms Involved in the Control of Tuberculosis

**DOI:** 10.1155/2012/193923

**Published:** 2012-05-17

**Authors:** Joaquin Zuñiga, Diana Torres-García, Teresa Santos-Mendoza, Tatiana S. Rodriguez-Reyna, Julio Granados, Edmond J. Yunis

**Affiliations:** ^1^Laboratory of Immunobiology and Genetics, Instituto Nacional de Enfermedades Respiratorias Ismael Cosío Villegas, Tlalpan 4502, 14080 Mexico City, DF, Mexico; ^2^Department of Immunology and Rheumatology, Instituto Nacional de Ciencias Médicas y Nutrición Salvador Zubirán, Vasco de Quiroga 15, Tlalpan, 14000 Mexico City, DF, Mexico; ^3^Department of Transplants, Instituto Nacional de Ciencias Médicas y Nutrición Salvador Zubirán, Vasco de Quiroga 15, 14000 Tlalpan, Mexico City, DF, Mexico; ^4^Department of Pathology, The Brigham and Women's Hospital and Department of Cancer Immunology and AIDS, Dana Farber Cancer Institute, Harvard Medical School, Boston, MA 02115, USA

## Abstract

*Mycobacterium tuberculosis* (Mtb) infection is a major international public health problem. One-third of the world's population is thought to have latent tuberculosis, a condition where individuals are infected by the intracellular bacteria without active disease but are at risk for reactivation, if their immune system fails. Here, we discuss the role of nonspecific inflammatory responses mediated by cytokines and chemokines induced by interaction of innate receptors expressed in macrophages and dendritic cells (DCs). We also review current information regarding the importance of several cytokines including IL-17/IL-23 in the development of protective cellular and antibody-mediated protective responses against Mtb and their influence in containment of the infection. Finally, in this paper, emphasis is placed on the mechanisms of failure of Mtb control, including the immune dysregulation induced by the treatment with biological drugs in different autoimmune diseases. Further functional studies, focused on the mechanisms involved in the early host-Mtb interactions and the interplay between host innate and acquired immunity against Mtb, may be helpful to improve the understanding of protective responses in the lung and in the development of novel therapeutic and prophylactic tools in TB.

## 1. Introduction

The contagious nature of tuberculosis (TB) was first known and described by Hippocrates and Galen. In the mid-seventeenth century, Sylvius (François de le Boë) and René Théophile-Hyacinthe Laennec described the tubercle and postulated that the tubercle was a common structure detected in all forms of the disease [1]. However, despite that the etiological agent of the disease had not been identified, the term “tuberculosis” was first used by Shönlein in 1830 [2]. Fifty-two years later, in March 1882, Robert Koch reported the discovery of the etiological agent that causes TB (the tubercle bacillus) [1]. Probably this is one of the most important events in the history of medicine. 

Despite the scientific advances applied to the diagnosis and treatment of* Mycobacterium tuberculosis* (Mtb) infection, it remains a significant public health threat, particularly in developing countries. One-third of the world's population is thought to have latent Mtb infection [3], a condition where individuals are infected by the intracellular bacteria without exhibiting the active disease but are at risk for reactivation. According to WHO reports, 1.4 million of deaths were associated with TB in 2010 worldwide [4]. The incidence of TB during 2010 worldwide was 8.8 million cases (128 cases/100,000 inhabitants), 59% of these cases were detected in Asia and 26% in Africa. In Mexico, the reported incidence was 18 cases/100,000 inhabitants, and 15,384 new cases were diagnosed during 2010 [4].

It is generally accepted that primary infections with Mtb produce active disease in approximately 10% of those infected [5]. In the majority of the infected persons, Mtb exists in the latent state, contained in the lungs, within structures called granulomas. In this regard, host immune mechanisms are involved in preventing the disease progression, and immune-compromised individuals are at a higher risk of reactivating the disease [5, 6].

In this review, we will focus on the progress that has been done in the host-Mtb interactions and the understanding of innate and adaptive cellular and humoral mechanisms involved in the pathogenesis of TB. We emphasize on Mtb-host immunity interactions in a sequential list of events trying to show a broad picture of the immunological aspects involved in the pathogenesis of active TB. In this context, we will discuss the role of toll-like receptors (TLRs) and the connection with adaptive responses as central mechanisms of protection from Mtb dissemination. The importance of macrophages, dendritic cells (DCs), and IFN-*γ* producing T cells in the early control of the Mtb infection and the production of other regulatory and proinflammatory cytokines such as IL-6, TNF-*α*, IL-12, and IL-23/IL-17 are also reviewed [7–10]. In addition, we discuss recent findings of the role of *γ*/*δ*T and Th17 cells in the protective immune response against the mycobacterial infections [11, 12]. We also review recent literature of mechanisms that contribute to the lack of Mtb infection control including mechanisms of Mtb evasion of immune response, immune dysregulation induced by autoimmune diseases and, the treatment with anti-TNF-*α* drugs and mechanisms of immunological escape inherent to the Mtb.

## 2. Pathogenesis of Tuberculosis

Based on experimental models, four events are well defined in the pathogenesis of pulmonary TB [13, 14].

**(A) Inhalation of the Mtb**

The early events following inhalation of Mtb involve the engulfment of the bacilli by alveolar macrophages and often their immediate killing by different macrophage bactericidal mechanisms, including the generation of reactive nitrogen intermediates (RNI) and reactive oxygen intermediates (ROI). The efficacy of these mechanisms depends on the intrinsic microbicidal capacity of the alveolar macrophages, the pathogenic characteristics of the inhaled Mtb strain, and the inflammatory microenvironment at the site of infection [15].

**(B) Inflammatory Cell Recruitment**

Bacilli which survive proliferate logarithmically within alveolar macrophages and DCs and induce the production of immune mediators such as TNF-*α*, IL-6, IL-12p80, IL-1*α*, and IL-1*β* that activate macrophages to induce early bacterial killing [5, 16]. IFN-*γ* is a proinflammatory cytokine produced by CD4+ and CD8+ T cells as well as by activated NK cells in response to IL-12 and IL-18 produced by alveolar macrophages and DCs [15, 17–19]. In a local lung inflammatory scenario induced by the proliferation of Mtb, peripheral inflammatory cells, including monocytes, neutrophils, and DCs, are recruited to the lung [20, 21]. DCs are activated trough TLRs signaling, and monocytes become differentiated to effector macrophages that produce microbicidal substances including TNF-*α*, that contributes to the control of Mtb growth, and granuloma formation [22].

**(C) Control of Mycobacteria Proliferation**

This phase is characterized by the inhibition of the Mtb proliferation with an efficient cell-cell interaction and the formation of a granuloma. As a result of chronic cytokine stimulation, macrophages differentiate into epitheloid cells and become fused giant cells [23]. The architecture of the granuloma is characterized by the aggregation of T cells and infected macrophages which contain the Mtb preventing their spread [23–25]. In addition to the key role of proinflammatory cytokines (e.g., IFN-*γ*, TNF-*α*, IL-6, IL-12, IL-17, and IL-23) in the formation and stability of the granuloma, the presence of chemokines such as CCL2, CCL3, CCL5, CXCL8, and CXCL10 is crucial for the recruitment of inflammatory cells to form granulomas [23, 26–28]. These mechanisms allow the development of a localized primary TB infection which eventually may become a stable (also known as a latent) infection. In more than 90% of the latent infections, the central caseous infectious foci containing live Mtb is delimited by the granuloma walls. An active cycle of cellular activation and suppression prevents the replication and spreading of the Mtb [5, 29].

**(D) Post-Primary Tuberculosis**

**Figure 1:**
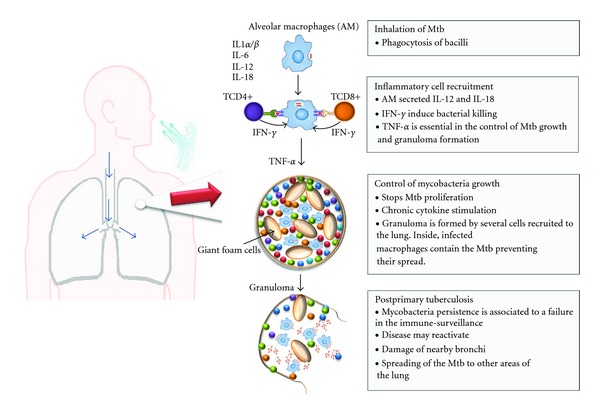
*Pathogenesis of tuberculosis.* TB pathogenesis can be divided in four well-defined events. Inhalation of the mycobacteria is followed by its interaction with resident macrophages through cellular receptors and its internalization. Macrophage bactericidal mechanisms are then activated, including RNI and ROI generation. The efficient killing of mycobacteria depends on pathogen and host factors. *Inflammatory cell recruitment*: survived mycobacteria proliferate within macrophages inducing the production of proinflammatory cytokines. The local inflammatory environment induces the recruitment of several cell types including monocytes, neutrophils, and dendritic cells to the site of infection. High levels of TNF-*α* contribute to control Mtb growth and granuloma formation. *Control of mycobacteria proliferation*: arrival of immune cells to the site of infection including T cells, which become organized in characteristic structures called granulomas efficiently stop mycobacteria proliferation and contain the mycobacteria within the granuloma walls preventing its spread. Characteristic of this structure is the presence of foam cells resulting from the differentiation of chronically activated macrophages. Mycobacteria containment eventually becomes stable (latent) infection. *Postprimary TB*: mycobacteria persistence associated with a failure in the immunosurveillance system increases the risk that latent disease becomes reactivated, inducing the damage of nearby bronchi and conditioning the spreading of the Mtb to other areas of the lung and the transmission of the disease.

As a result of the mycobacteria persistence, associated with a failure in the immunosurveillance system, latent disease may be reactivated, inducing the damage of nearby bronchi and conditioning the spreading of the Mtb to other areas of the lung [30], Figure 1.

## 3. Role of TLRs in the Control of Mtb Infection

The control of Mtb infection begins with the recognition of mycobacterial structural components of the cell wall such as mycolic acids, peptidoglycans, arabinogalactins, phosphatidyl-myo-inositol mannosides (PIM), mannose-capped lipoarabinomannan (Man-LAM), lipomannan, and mannoglycoproteins [31, 32]. The recognition of conserved molecular patterns is possible through pattern recognition receptors (PRR), among them the best known are the TLRs, nucleotide-binding oligomerization domain-like receptor (NLR), C-type lectins, and scavenger and complement receptors [33–36].

The interaction of Mtb conserved molecular patterns with the PRR triggers the activation of diverse innate immunity mediators involved in the phagocytosis of mycobacteria and signaling pathways related to the IL-12, TNF-*α*, and IL-1*β* production [36, 37].

**Figure 2:**
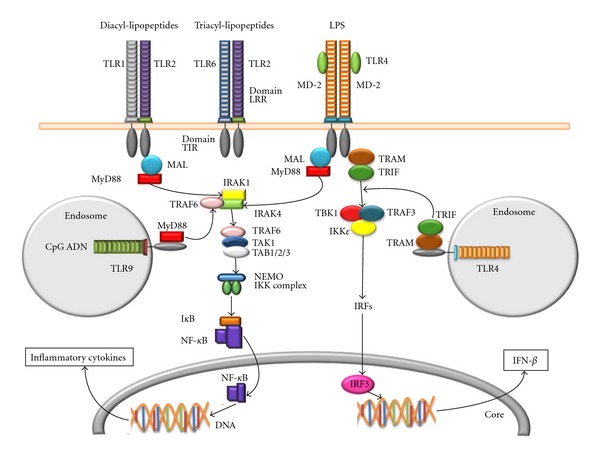
TLRs signaling pathways involved in the recognition of Mtb structures.TLR receptors can recognize several conserved molecular patterns of the mycobacteria cell wall. Diacylated or triacylated proteins as well as LPS are recognized by membrane receptors (TLR1, TLR2, TLR6, and TLR4), whereas bacterial unmethylated CpG DNA can be recognized by endosomal TLR9. MyD88 adaptor is a central component in TLR signaling whose downstream signaling cascade leads to the activation of NF-kB and AP-1 transcription factors and to the production of inflammatory cytokines. Signaling through TRIF adaptor molecule activates IRF-3 transcription factor inducing IFN-*β* secretion.

TLRs are a family of phylogenetically conserved genes which are essential for recognition of a broad repertoire of microbes on macrophages and DCs. It is well accepted that TLR2, TLR4, and TLR9 play an important role in the innate responses against Mtb [38–41], Figure 2.

TLR2 forms dimers with TLR1 or TLR6 which recognize diacylated or triacylated lipoproteins. In addition, lipoarabinomannan (LAM), lipomannan (LM), and phosphatidylinositol mannoside (PIM) from Mtb may also be recognized by TLR2 [22, 41]. Importantly, despite *in vitro* evidence that TLR2 is important in the control of several aspects of effector functions of antigen presenting cells, it has been demonstrated that TLR2 deficiency in experimental models does not affect the development of secondary immune responses to Mtb [42–46].

The cell wall of gram-negative bacteria constituent lipopolysaccharide (LPS), a potent proinflammatory pathogen-associated molecular pattern, is the ligand for TLR4 receptor, Figure 2. LPS recognition by TLR4 induces the expression of NF-kB and regulates the production of proinflammatory cytokines and chemokines including IL-1, IL-6, and CXCL8 [39, 47–50].

The critical role of TLR4 in the development of efficient innate host responses to mycobacteria has been demonstrated in functional studies in mice. TLR4-deficient C3H/HeJ mice are more susceptible to develop lethal Mtb-infection compared with normal C3H/OuJ mice [47]. Furthermore, the expression of TNF-*α*, IL-12p40, and CCL2 in lung and in Mtb infected cultures from TLR4-deficient C3H/HeJ mice is impaired when compared with normal mice [48].

The importance of TLR9 in the recognition of bacterial unmethylated CpG motifs has been also demonstrated in TLR9-deficient mice [37, 38, 51]. *In vitro* studies have confirmed that stimulation through TLR9 induces IL-12 production by DCs [38]. On the other hand, conflicting evidence from experimental studies has shown that macrophages obtained from triple knockout mice TLR2-TLR4-TLR9 display similar capacity to the wild type mice to control Mtb [38].

The variability in the outcome of Mtb infection in single, double, or triple TLR2-, TLR4-, and TLR9-deficient mice may be due to differences in the Mtb strains used or due to differences in the genetic background and functional immunity of mice strains used in different studies. Contrasting with the controversial results on the role of TLRs in the innate control of Mtb infection, a growing body of evidence supports the essential role of the myeloid differentiation factor 88 (MyD88) in the development of protective responses against Mtb [52, 53]. MyD88 is a key signaling adapter in TLRs signaling [52, 53]; MyD88 binds to the cytoplasmic portion of TLRs and associates with IRAK-4 and IRAK-1 through the interaction of their death domains. IRAK-4 phosphorylates and activates IRAK-1 that in turn associates with TRAF6 leading to its oligomerization and activation. Posterior binding of Ubc13 and Uev1A promotes TRAF6 ubiquitination, TAB2 recruitment, and TAK1 activation assembling a larger complex leading to the activation of the transcription factors NF-kB and AP-1 [22, 54], Figure 2. MyD88-deficient mice are highly susceptible to infection by several pathogens including Mtb [55–58].

Recent evidence suggests that interaction of early secreted antigenic target protein 6 (ESAT-6) with TLR-2/MyD88 efficiently induces the differentiation of Th17 cell protective responses against Mtb [59]. In addition, MyD88 is also involved in the signaling cascades from receptors of IL-1*β* and IL-18 [60–62]. In this context, mice deficient for IL-18 promptly develop a lethal H37Rv strain Mtb infection when compared with wild-type and TLR2-TLR4 double knockout mice. IL-18-deficient mice also exhibit a significant impaired Th1 response [63].

In addition to the TLRs, other mechanisms of innate immunity that are not discussed in this review such as NOD2- and NOD-like receptors, C-type lectin receptors, multiprotein complexes also called inflammasomes, and vitamin D have been extensively studied [64].

## 4. Cytokines and Chemokines Produced in Response to Mtb Infection

Chemokines are a large family of structurally related proteins that regulate inflammation, immune cell trafficking, and differentiation through their interaction with 7-transmembrane G-protein coupled receptors. Chemokines have been grouped according to structural similarities and the presence of conserved cysteine residues. The chemokines with two of four consecutive cysteine residues are known as CC chemokines, whereas CXC chemokines have one aminoacid between the first two of four cysteine residues. C chemokines, with one cysteine, at the amino terminus and CX3C chemokines containing three intervening amino acids have been described. The receptors are designated by the chemokine class to which it binds, followed by the letter “R” and a chronological number [65]. The contribution of chemokines in the control of Mtb infection has been supported by several *in vitro* and *in vivo* studies [20, 66, 67]. Once the macrophage engulfs Mtb, it produces several cytokines and chemokines which induce the development of proinflammatory responses. Mtb infection of macrophages induces the production of various chemokines including CCL2, CCL3, CCL5, CCL7, CCL12, CXCL2, CXCL8, and CXCL10 [67]. These chemokines are closely related with activation of microbicidal responses promoting the migration of different cell subpopulations to the Mtb-infected tissues to form granulomas [68]. Several studies have investigated the effects of chemokines in the function and recruitment of monocytes following the infection with Mtb. They promote monocytes, DCs, activated macrophages, polymorphonuclear cells (particularly neutrophils), and T lymphocytes migration to bronchoalveolar spaces during pulmonary TB [69].

Inflammatory monocytes infiltrates are significantly reduced in CCR2-deficient mice infected with Mtb. During TB infection in normal mice, recruited monocytes express CCR2 which has different agonists, CCL2, among them [70]. In line with these observations, it is accepted that CCL2 is a central activator of macrophages. Secreted chemokines play a significant role in the recruitment of effector T cells to the site of Mtb infection [71]. *In vitro* analysis has demonstrated that TLRs (TLR1, TLR2, TLR3, TLR4, and TLR-9) are relevant in the signaling for CCL2 induction through different transcription factors including NF-kB and MAP kinases [72, 73]. A direct correlation between elevated CCL2 levels in TB and severity of the disease has been recently reported [74]. Importantly, CCL2 exerts functional activity in the recruitment of both Th1 and Th2 cells and facilitates the polarization of naïve T cells to Th2 cells as a result of IL-4 upregulation. In this perspective, increased CCL2 levels may promote an excessive polarization to Th2 responses, resulting in a defective control of Mtb infection [75–77].

CCL5 is a chemokine produced by a variety of cells including macrophages, fibroblasts, eosinophils, endothelial cells, and platelets. CCL5 exerts chemotactic activity on DCs, T lymphocytes, polymorphonuclear cells, NK cells, and mast cells to inflamed or infected tissues. The expression and functional activities of CCL5 have been studied in experimental models of infection with mycobacteria. CCL5 blockade affects the recruitment of cells and the formation of granulomas induced by *M. bovis* antigens [78, 79]. CCL5 is important in early responses to Mtb due to its role in the recruitment of IFN-*γ* producing T cells to form lymphocyte-enriched granulomas [79]. In contrast, there is one study that suggested that CCR5 and their ligands (including CCL5) are not essential to the development of protective responses to Mtb. In this study, significant differences in the response to the pathogenic H37RV strain of Mtb between CCR5-deficient mice and wild-type mice were not detected [80].

The efficient induction of Th1 immunity is decisive for the defense against Mtb. The classical cytokines produced in response to Mtb infection are IL-2, IFN-*γ*, IL-6, IL-1*α*/*β*, IL-12, and TNF-*α* [17, 18, 81–84].

### 4.1. TNF-*α*

This cytokine mediates early inflammatory responses against pathogens and is produced by a variety of cells including macrophages, lymphocytes, neutrophils, mast cells, and endothelial cells. Several functions have been attributed to TNF-*α*, but probably one of the most relevant functions is the regulation of the inflammatory response, stimulating the production of IL-1 and IL-6 [15]. The importance of TNF-*α* in the defense against Mtb was established by the increased susceptibility to BCG infection in mice treated with anti-TNF-*α* antibodies [85, 86]. The mechanisms of Mtb susceptibility associated with the use of TNF blockers will be discussed in one of the next sections of this review. TNF-*α* contributes in the control of Mtb infection by the induction of RNIs and ROIs by macrophages and early induction of chemokines. TNF-*α*-deficient mice produce reduced levels of chemokines resulting in defective granuloma formation [87].

### 4.2. IFN-*γ*

The function of IFN-*γ* in response to pathogens has been extensively studied and is critical in the regulation of T cell responses in mycobacterial disease [9]. IFN-*γ* is produced by activated T cells, NK cells, and macrophages, and it is essential for the activation of phagocytes and antigen presentation, and it promotes cellular proliferation, cell adhesion, and apoptosis. In macrophages, IFN-*γ* induces respiratory burst contributing to the production of RNIs and ROIs [9]. In addition, activated macrophages produce immunomodulatory and chemotactic molecules that promote upregulation of TNF-*α* receptor and NRAMP-1. The production of large amounts of ROIs and NO by innate immune cells is considered one of the most important effects of IFN-*γ*. In the mice model of Mtb infection, NO is essential in the killing of Mtb by mononuclear phagocytes. In NO synthase 2 gene-deficient mice (iNOS−/−), the infection with Mtb has a rapid progression and a higher rate of mortality [88, 89]. Marked defects in the IFN-*γ*-signalling pathways, observed in IFN-*γ* deficient mice, provoke high susceptibility to mycobacterial infections. These mice also fail to develop granulomas following aerosol Mtb infection with a significant impairment of macrophage activation [90].

### 4.3. IL-6

It is a cytokine with a wide variety of functions that can exert pro- or anti-inflammatory effects and it is critical to the development of early inflammatory mechanisms. IL-6 is also involved in the development of T and B lymphocyte responses and hematopoiesis and is secreted by different cell types, including B and T cells, phagocytes, fibroblasts, and endothelial cells [91–93]. IL-6 is produced in response to Mtb in early phases of the infection. The absence of this cytokine in the low dose of Mtb mice model infection promotes a delayed IFN-*γ* response in the lung and a slight increase in the Mtb burden [94, 95].

The evidence supports the idea that IL-6 is critical in the modulation and maintenance of the IL-17-producing cells in response to Mtb infection in mice [96]. In addition, IL-6 and IL-12 are critical in the development of efficient T lymphocyte antimycobacterial responses, and IFN-*γ*-mediated responses against Mtb following vaccination with the short-term-culture filtrate (ST-CF) antigens from Mtb [97].

### 4.4. IL-12

Mtb efficiently promotes the production of IL-12p40 subunit. IL-12 is a cytokine that promotes the development of Th1 responses and is rapidly produced by DCs through the interaction of Mtb with TLRs. Interestingly, some studies have suggested that induction of IL-12 production is dependent on TLR9 in DCs and that it is dependent on TLR2 in macrophages [98]. Importantly, it has been demonstrated that murine macrophages release significantly lower IL-12 amounts than DCs in response to Mtb infection [99, 100]. IL-12 deficiency increases the susceptibility to mycobacterial disease in humans [101].

### 4.5. IL-23/IL17

Recent studies have demonstrated that the IL-23/IL-17 pathway may have a crucial role in the immunity against several pathogens, particularly in mycobacterial infection [26, 102]. These cytokines have been involved in the development of protective and regulatory immune responses in mice and humans infected with Mtb. The role of IL-17 in the development of antimicrobial responses, chemokine production, and recruitment of inflammatory cells for control of pathogens has been described in several studies [27, 103–105]. In *M. bovis* BCG infection, the absence of IL-17 does not influence the overall survival or susceptibility to infection but has an effect in the formation of granulomas in the lung [106]. In consonance, recent findings indicate that IL-23 is not determinant for the early control of Mtb infection but is required for the development and maintenance of Th17 responses to Mtb [107].

## 5. T Cell Responses in TB

As we mentioned before, the control of Mtb infection in the lung depends on the development of efficient innate and adaptive responses. CD4+, CD8+, and *γδ*T lymphocytes play an important role in the protection from Mtb [15]. CD4+ T cells produce immune mediators (IFN-*γ*, IL-2, and lymphotoxin alpha) [108] CD8+ T cells, and *γδ*T cells release granzymes and perforins that exert a direct effect on mycobacteria and infected cells through Fas ligand dependent and independent mechanisms. Peripheral T cells recognize Mtb antigens (ESAT-6 and Mtb-39) which stimulate the production of IFN-*γ* and TNF-*α* [12, 17, 109]

It has been established that the development of effector T cell responses requires the dissemination of live bacteria or bacterial antigens presented by DCs that upregulate the expression of CCR7 and migrate to the draining lymph nodes [110, 111]. It is possible that other cell types also contribute in the transport of Mtb antigens to the lymph nodes. In this regard, some studies have suggested that neutrophils transport live BCG to draining lymph nodes after intradermal vaccination [112]. The proliferation of naïve Mtb-specific T cells occurs first in the draining lymph node in response to the dissemination of Mtb [113]. Once the antigen-specific T cells become activated through the presentation of Mtb antigens on MHC class I and class II on the surface of antigen-presenting cells, they migrate to the primary areas of infection in the lung 15–18 days after infection [114]. Mtb infection might affect the migration of DCs to the draining lymph nodes by the induction of IL-10 production [115]. Interestingly, high numbers of antigen-specific CD4+ CD69+ Th1 cells against ESAT-6 and CFP-10 peptides have been detected in pleural fluid from patients with pleural TB. In this study, the authors suggest that based on the expression of CD69 on Th1 antigen specific cells, CD69 is a useful marker to identify antigen specific Th1 responses in patients with TB [116].

The development of efficient memory CD4+ and CD8+ T cell responses is the main goal of the novel vaccine strategies against Mtb. In addition to the importance of CD4+ and CD8+ T cells in TB, a growing body of evidence supports the importance of *γδ*T cells in the cellular immunity against mycobacterial infections. Recent reports have shown that patients with active TB have reduced levels of V*γ*9/V*δ*2 T cells when compared with healthy donors [11]. The mechanisms explaining how *γδ*T cells influence the innate or acquired responses against Mtb are not completely understood. Nevertheless, *γδ*T lymphocytes are a major source of IL-17 and IFN-*γ*, key cytokines in the proinflammatory responses and chemokine regulation. In mice, the absence of IL-17 provokes a significant reduction in the mononuclear and polymorphonuclear inflammatory infiltrates to the lung [26, 117, 118]. In our published studies, overexpression of IL-17 was found in peripheral mononuclear cells from latent tuberculosis individuals [119].

These findings need to be explained in regards to the controversy related to the discrepancies found in the Th1/Th2 paradigm [120]. In addition, the studies of the influence of microbiome diversity in the driving of immunity against Mtb could be helpful to understand the variability of innate and acquired responses, particularly Th1 or Th2 phenotypes observed in Mtb-exposed individuals [121, 122].

Several studies have supported the role of IL-23 and IL-17 in the formation, maintenance, and long-term integrity of granuloma in advanced stages of the inflammatory process induced by Mtb. Differentiation of Th17 cells depends on the production of IL-23, IL-1*β*, IL-6, and TNF-*α*. The importance of Th17 cells is illustrated by the fact that defective production of Th17-related cytokines influences the outcome of the infection [117, 123–127].

Human natural killer (NK) cells are important in the innate defense against pathogens [128], and they also mediate antimycobacterial activity. NK cells express surface-binding proteins that have the capacity to bind ligands on the surface of most cells. The interplay between stimulatory and inhibitory signals determines the functional activation of NK cells. Previous studies have demonstrated that human NK cells can recognize and lyse macrophages infected with Mtb using the NKp46 receptor [129]. It has been demonstrated that once the human macrophages become infected, they upregulate vimentin, and NK cells can lyse them via ligation of the vimentin with the activator NKp46 receptor [130]. In addition to the role of activated NK cells in the lysis of infected cells, they also produce significant amounts of IFN-*γ*, IL-22, and other cytokines. A direct effect of IL-22 produced by IL-15 and DAP-10-stimulated NK cells in the control of Mtb proliferation has been described [131].

## 6. Humoral Mechanisms Possibly Involved in Latent Tuberculosis Infection

 We have summarized the accepted evidence that innate and adaptive cell-mediated immunity (CMI) plays a pivotal role in the response to Mtb infection in experimental models and humans. In this section, we will discuss published pertaining evidence about the role of humoral immunity in the protection and control of Mtb infection. Supportive information about the importance of antibodies in the host defense against mycobacterial infections has been provided by experimental studies in both animals and humans which demonstrate that specific antibodies neutralize pathogen toxins, promote opsonization, and modulate complement-mediated lysis. Several authors have reported the use of ELISA assays to measure titers of IgG specific to mycobacterial antigens in adult patients with pulmonary TB [132] and children with disseminated TB [133]. In the first study of adults with TB, those patients who received anti-TB treatment for 4 weeks or more exhibited higher IgG titers when compared with patients who received less than 4-week treatment. Furthermore, children with disseminated TB had lower levels of IgG against mycobacterial antigens than children with localized infection. In addition, the levels of IgG anti-PPD antibodies in TB-HIV patients varies with the stage of HIV infection, indicating a progressive impairment of the antibody responses in advanced stages of HIV infection [134]. Another study, in HIV-TB coinfected individuals, reported significant differences in the titers of LAM-specific IgG1, IgG2, and IgG4 subclasses between HIV-TB and TB patients. Interestingly, serum levels of anti-LAM antibodies were not detected in HIV patients without TB, suggesting that these antibodies participate in protective immune responses against TB in these individuals [135]. Our experiments of *in vitro* cultures of human PBMCs stimulated with tuberculin comparing the role of autologous sera in active TB patients (ATBP) demonstrated that the levels of IgG antituberculin antibodies correlate with blocking of the proliferation responses to tuberculin [136]. Such results suggested that the polarity of responses could be produced by either Th1 or Th2 immunity. In this regard, two different forms of leprosy correlate with Th1 or Th2 polarity of the immune response [137, 138]. In tuberculoid leprosy, patients mount a strong Th1-type cell-mediated immune-response producing interferon-*γ* and IL-2 in lesions and localize the infection, restricting the growth of the bacteria. While in lepromatous leprosy, a widely disseminated form of the infection is associated with a Th2 cytokine response with decreased cell-mediated immunity [139]. It remains to be determined if the same polarity exists in high-risk latent TB (LTBI) contacts that produce high levels of antituberculin IgG antibodies [136, 140, 141]. The absence of a positive tuberculin skin test (TST) has also been described in immunocompetent anergic patients diagnosed with active TB, and the inability to mount an antigen-specific delayed-type hypersensitivity (DTH) response to PPD was shown to be associated with a defective T cell response including an antigen-specific impaired ability to produce IL-2 and to proliferate in response to the PPD [136, 142] regardless of prior BCG vaccination status [143]. These findings could be due to a defective phosphorylation of T cell receptor (TCR) leading to a defective activation of ZAP-70 and MAPK proteins related to IL-2 gene transcription and protein production after antigen stimulation of T cells [144]. Additionally, a decrease of the *γδ*T cells subset [11], a decrease of IFN-*γ* production, an increase of IL-10 production in response to PPD [145], and a failure of the autophagic control by macrophages have been associated with anergy in ATBI [146]. In previous studies, we analyzed the antibody profiles to explain the presence of false negative or anergy to TST responses in high-risk individuals without active TB infection. Our results showed that regardless of the result of the TST, antituberculin antibody titers correlate with the protection from active TB and that the presence of high antituberculin antibody titers is a reliable indicator of latent infection. Importantly, high levels of antituberculin isotype IgG3 antibodies could prevent the reactivation of the disease in high-risk individuals [147].

In addition, studies in Mexican-Amerindian ethnic groups have reported that IgG anti-Ag85 antibodies had beneficial effects in the clinical outcome of pulmonary tuberculosis [148]. 

Studies in mice have demonstrated that immunotherapy with monoclonal antibodies specific to anti-Mtb surface antigens reduces the systemic infection and prolongs survival of mice infected with Mtb [149, 150].

Furthermore, recent studies demonstrate that passive serum therapy with human polyspecific IgG protects mice from Mtb Infection [151].

Recent findings demonstrated that intranasal inoculation of human IgA specific to the mycobacterial *α*-crystallin antigen significantly reduces the infection with the H37Rv Mtb strain in CD89 transgenic mice [152].

Several mechanisms have been proposed by which antibodies could mediate protective effects against mycobacterial infections. These mechanisms include the interference of mycobacterial adhesion to cells possibly mediated by antibodies specific to surface structural antigens of Mtb, limiting the establishment or dissemination of Mtb infection to other cells or tissues. The interference of adhesion mediated by antibodies was demonstrated in experimental assays in which the preincubation of Mtb with anti-LAM antibodies inhibited the attachment to human macrophages [153]. Other proposed mechanisms are the enhancing of phagosome-lysosome fusion [154], bacterial products or toxins neutralization, and cellular- or complement-mediated bacterial lysis [154].

We consider that it is relevant to develop experimental strategies to understand the role of humoral responses in the outcome of mycobacterial infections. Functional studies in both human and animals have revealed significant results about the protective effect of antibodies. Finally, the studies of passive immunotherapy using monoclonal antibodies against specific Mtb antigens in transgenic mice are critical to understand the molecular mechanisms that promote antibody-mediated protection.

It is important to mention that the BCG vaccine developed one century ago remains one of the most widely used vaccines. However, with the exemption of tuberculosis meningitis in children, its capacity to protect the population against Mtb infection is controversial because randomized trials reported an important variation of protection from zero to 80% [155]. All attempts to replace BCG have achieved poor success due to our incomplete knowledge of mechanisms of Mtb immunity.

## 7. Factors That Contribute to the Lack of Control of Mtb Infection

### 7.1. Mechanisms of Mtb Evasion

Pathogens like Mtb have evolved complex mechanisms to evade, divert, or subvert immune responses. Mtb is one of the most successful human pathogens. Different from other pathogens, Mtb infection can persist in the host for long periods in a dormant or latent state, even in a fully functioning immune system. TB immunity is mediated by Th1-type responses, nevertheless this response is apparently not enough to definitively eradicate the chronic infection nor the latent bacilli. Within the strategies of Mtb to evade or subvert immune responses, the best documented mechanisms are those that avoid the elimination of the bacilli within macrophages and those that interfere with an adequate antigen presentation for T cell activation.

Macrophages are the major cell type infected by Mtb *in vivo*, and they are the site of intracellular replication of the mycobacteria. It is well documented that Mtb inhibits the phagosome maturation and its fusion with the lysosome in infected macrophages [156]. Mycobacterial phagosomes retain GTPase markers of early endosomes such as Rab5, and they limit the acquisition of late endosome markers such as Rab7, leading to the arrest of phagosome maturation [157]. In addition, some studies show that mycobacterial phagosomes failed to acquire the vacuolar H+-ATPase that is necessary for the phagosome acidification [158]. Retention of the tryptophan aspartate-rich coat protein (TACO) in the surface of mycobacteria-containing phagosomes also participates in the inhibition of the lysosome fusion with the phagosome, which avoids the destruction of mycobacteria by the lysosomal enzymes [159, 160]. As we mentioned before, NO and RNIs are potent antimycobacterial species produced within macrophages by the activity of NOS2. Mtb has a sophisticated mechanism in which several enzymes such as peroxiredoxin alkyl hydroperoxide reductase subunit C (AhpC), dihydrolipoamide dehydrogenase (Lpd) dihydrolipoamide succinyltransferase (SucB), or thioredoxin-like AhpD are complexed to constitute a nicotinamide-adenine-dinucleotide (reduced-) dependent peroxidase and peroxynitrite reductase, generating an antioxidant defense that confers RNIs resistance [161]. All these mechanisms account for the survival and propagation of mycobacteria within infected macrophages.

Another survival strategy of Mtb within the host is to prevent the recognition of infected macrophages by CD4+ T cells, inhibiting MHC class II processing and presentation. It has been described that the 19KDa lipoprotein antigen of Mtb induces the downregulation of MHC class II molecule expression in infected macrophages presumably due to the excessive TLR2 stimulation. Since TLR2 is not only present in the surface of the macrophage but also present within the phagosome, it seems that chronically infected macrophages constantly downregulate MHC class II molecules by intraphagosomal TRL2 stimulation [42, 162, 163]. The IL-10 secretion from infected macrophages inhibits cathepsin S, a protease necessary for the invariant chain degradation and the proper loading of class II molecules and subsequent surface expression [164].

An interesting emerging theme is the potential regulation of autophagy during Mtb infection. Autophagy is a homeostatic mechanism that involves the autodigestion and turnover of long-lived cytoplasmic macromolecules and organelles [165, 166]. It has been demonstrated that physiological IFN*γ* or pharmacological induction of autophagy promotes mycobacterial phagosome maturation and inhibition of mycobacterial survival in infected macrophages [166]. More recently, the mycobacterial gene *eis* (enhanced intracellular bacterial survival) has been implicated in the inhibition of autophagy in infected macrophages [167]. The comprehensive mechanisms of autophagy and its potential contribution to the regulation of Mtb infection warrant further investigation.

Recent studies have demonstrated that DCs are also infected with Mtb during mice experimental infection [168]. Mice airway immunization induces a delay in the expression of costimulatory molecules (maturation) in DC and a delay in the migration to the mediastinal lymph nodes (MLN) where they begin to appear 14 days after immunization and peak at day 21, in stark contrast to other soluble or particulate antigens administered through the airways, where mature DCs arrive to the MLN 24–48 h after immunization. This suggests that mycobacteria exert mechanisms that actively inhibit DC functions and the onset of adaptive immune responses [168, 169].

The relevant participation of Th1-type immune response in the control of Mtb infection is clearly evident in the altered genetic pathways for both the IFN*γ* and IL-12 axis [170]. Mtb has developed many strategies to overcome this adaptive immune response. Data from many laboratories have documented the delay in the onset of adaptive immune response against Mtb both in mice model and in humans [171], nevertheless, the exact mechanisms that explain this delayed response are not well understood.

Specific CD4+ T cells are primed by DCs in the MLN, then they proliferate and migrate to the lung to eliminate the mycobacteria [114]. Unlike the T cell involvement in infections with other pathogens that enter through the airways, in Mtb infection the specific T cells begin to appear in the lung 14 days after aerosol infection [113]. This is particularly intriguing since systemic peripheral response by means of DTH assays is clearly seen 3 days after inoculation, meaning that a delayed adaptive immune response is specific of the Mtb target organ, the lung [168, 171]. In addition to the delay in the onset of specific T cell response in the lung, the presence of specific T cells with inefficient activation phenotype or exhaustion phenotype has been described [172, 173]. Airborne infection of mice with the virulent strain H37Rv triggered a considerable and progressive increase in PD-1 positive T cells, both in the MLN and the lung [174]. Moreover, peripheral blood or pleural fluid from Mtb-infected individuals also contained high numbers of PD-1+ T cells [173]. In addition, a mice model suggests that the percentage of effector IFN-*γ* producing CD4+ specific T cells in the lung displays progressive decrease during infection that correlates with the downmodulation of antigen expression by mycobacteria [172]. It has been proposed that Mtb promotes a suboptimal effector function of CD4+ T cells as another evasion mechanism; however, future functional assays are needed to determine the possible mechanisms implicated in this phenomenon.

### 7.2. Tuberculosis in Patients with Inflammatory Immune-Mediated Diseases

Several epidemiologic studies have shown that rheumatoid arthritis (RA), systemic lupus erythematosus (SLE), and other autoimmune disease patients have an increased risk of bacterial and mycobacterial infections compared with the general population, possibly due to immune system innate or acquired defects, but certainly also due to the use of immunosuppressive drugs. In the clinical setting, it is difficult to separate the risk derived from the disease itself from the risk conferred by the use of immunosuppressive drugs, hence, all patients with autoimmune diseases require adequate TB prophylaxis and a high index of suspicion for TB infections because in these patients there is higher index of extrapulmonary TB (up to 52% in some case series), and the clinical picture may mimic a disease flare [175–177].

#### 7.2.1. Tumor Necrosis Factor Antagonists and Risk of *Mycobacterium tuberculosis* Infection

Tumor necrosis factor antagonists have been a major advancement in the treatment of several immune-mediated inflammatory diseases, including rheumatoid arthritis (RA), ankylosing spondylitis (AS), Crohn's disease, psoriatic arthritis, and psoriasis. These drugs include chimerical human-murine monoclonal antibodies (infliximab), fully human monoclonal antibodies (adalimumab, golimumab), pegylated recombinant humanized antibody Fab fragment against TNF (certolizumab), and a soluble dimeric fusion protein that acts as a TNF receptor (etanercept). They have been recognized as a risk factor for active tuberculosis, independently of the underlying disease, since it has been observed in patients with several inflammatory diseases [178]. Most of the available information is about patients with rheumatoid arthritis, in whom the risk of tuberculosis is 4–10-fold increased with the use of TNF antagonists [179–181]. The disease pattern in these patients is characterized by a high rate of extrapulmonary (56–62%) and disseminated disease (24–28%) [182].

The five currently FDA-approved TNF*α* antagonists differ substantially in their structure and pharmacokinetics [183]. These functional differences may have important implications for both their effectiveness and their adverse event profile. It is clear from several cohort studies that TB incidence rates are higher with the use of infliximab and adalimumab compared with etanercept, and despite screening and treatment guidelines, recent studies still show this tendency. In a French biotherapy registry, the standardized incidence ratio (SIR) was 18.6 TB cases for patients treated with infliximab, 29.3 TB cases for patients treated with adalimumab, and 1.8 TB cases for patients treated with etanercept [184]. In the British Society for Rheumatology Biologics Register, the rate of TB was higher for adalimumab (144 events/100,000 person/years) and infliximab (136/100,000 person/years) than for etanercept-treated patients (39/100,000 person/years) [181]. Golimumab and certolizumab seem to share the risk to develop TB. Although no comparative studies have been published yet, several TB cases have been reported in the clinical trials that have evaluated these drugs [185–187]. Several studies have revealed that most TB cases are detected within the first few months of the treatment with infliximab but at a stable rate throughout the treatment with etanercept, suggesting that TB cases associated with infliximab may be due to reactivation of latent disease, while TB cases associated with etanercept may be due to both reactivation and primary infection [181]. 

#### 7.2.2. Mechanisms That Lead to Increased Risk of Active Tuberculosis Infection

It is evident that TNF-*α* plays a relevant role in the defense against Mtb; therefore, it is expected that blockade of the effects of this molecule would alter substantially the immune response to Mtb. Many studies have demonstrated that TNF-*α* blockers have significant effects on various immune cells, both in their activation and responses. These effects have an impact on innate and adaptive immune responses to many microorganisms, including Mtb. Nevertheless, experimental studies that have explored the effects of anti-TNF drugs in the immunopathogenesis of TB have shown variable and even controversial results.

**(a) Effects on Phagosome Maturation and Autophagy**

There are experimental data that indicate that TNF blockers hamper phagosome maturation. Activation of macrophages with IFN-*γ* induces maturation and acidification of mycobacteria-containing phagosomes, leading to increased intracellular killing by macrophages *in vitro* [16, 188] Harris et al. showed that this IFN-*γ*-induced increase in phagosome maturation is inhibited in human macrophages exposed to the TNF blockers adalimumab and infliximab but not in those exposed to etanercept; in addition, monocyte-derived macrophage pretreatment with TNF induces phagosome maturation [189].

In previous sections of this review, we discussed the potential role of autophagy during Mtb infection. Physiological IFN-*γ* or pharmacological induction of autophagy promotes mycobacterial phagosome maturation and inhibition of mycobacterial survival in infected macrophages [166, 167]. Since, IFN-*γ*-induced phagosome maturation depends upon autocrine TNF secretion and there are some studies that suggest that TNF can induce autophagy [190–192], it is possible that TNF blockers may also inhibit autophagy.

**(b) Effects on T Cells.**

Several studies have explored the effects of TNF-*α* blockers on apoptosis on immune cells, showing variable results. There are studies that show that infliximab induces apoptosis in activated but not resting T cells both *in vitro* and *in vivo* in patients with Crohn's disease [193, 194].

In addition, CD8+ T cells, that mediate Mtb-infected-macrophage cell death by perforin and granulysin release, can die by complement- dependent cytotoxicity (CDC) when exposed to infliximab [195].

Other models using human Jurkat T cell lines that express transmembrane tumor necrosis factor (tmTNF) have shown that infliximab and adalimumab, increases the CDC, and infliximab, adalimumab and etanercept can induce antibody-dependent cell-mediated cytotoxicity (ADCC) when tmTNF-expressing Jurkat cells are added to lymphocytes isolated from peripheral blood [196, 197].

Saliu et al. [198] showed that activation of T cells can be modified by TNF blockers; they incubated whole blood from TST positive donors with infliximab and adalimumab and observed a significant decrease in the proportion of activated (CD69+) CD4+ T cells responding to Mtb and also a significant reduction in the antigen-induced secretion of IFN-*γ* and IL-10. Etanercept did not show similar results. A similar experiment by Hamdi et al. [199] showed that infliximab and adalimumab inhibited PBMC proliferation from patients with previous TB or LTBI in response to PPD and other antigens. In addition, in patients that showed normal proliferative or IFN*γ* responses to PPD, treatment with TNF blockers left PPD-specific proliferative response unaltered but significantly reduced the number of PPD-specific IFN*γ*-producing cells. The decreases were similar after treatment with infliximab or etanercept and were independent of the underlying disease (rheumatoid arthritis, spondyloarthropathy, or Crohn's disease). These results only partially explain the differences in the Mtb infection rates in patients treated with different TNF-*α*-blockers.

In contrast with these results, other studies have shown that treatment of patients with etanercept or infliximab can significantly enhance the proliferation and IFN-*γ* production of PPD-stimulated PBMC, including CD45RA CD4+ T cells. These controversial results may be explained, in part, by methodological differences, heterogeneity of the diseases, or the dual role of TNF-*α* in Mtb infection [200–203].

**(c) Effects on Dendritic Cells and Monocytes**

Etanercept can induce apoptosis of dermal DCs in plaques of psoriatic patients [204], while adalimumab and infliximab can induce apoptosis in lipopolysaccharide-stimulated human CD33+ blood monocytes [205].

## 8. Concluding Remarks

The efficient and dynamic interaction between innate and acquired immune responses is critical in the establishment of host-Mtb interaction and in the control of infection. Several cell types and molecules produced early in response to Mtb determine the establishment of the primary infection and the outcome of the disease after dissemination of Mtb. In this context, signaling mediated by a redundant interaction between Mtb and TLRs frequently induces an efficient innate activation of infected macrophages resulting in enhanced bacterial killing. However, future studies should be focused on the role of the TLRs and other innate receptors and the connection of innate and adaptive responses in order to clarify their role in the generation of latent TB states or in the outcome of clinically active Mtb infection. In addition to the innate and T-cell-mediated immunity, it is important to revisit the role of humoral immunity in the generation of antibody-mediated protective responses in Mtb infection. Transgenic mice with human immunomodulatory molecules should be used to prove the importance of immunotherapy in the control of mycobacterial infections. In this context, it is well known that Th-1 cells are highly proinflammatory and are known to be involved in the progression of autoimmune diseases, whereas Th2 are involved in allergy. There is no doubt that immunity to Mtb depends on Th1-cell activity (IFN-*γ* and IL-12 and the production of TNF-*α*), but Th1 immunity alone is not sufficient to protect the host from Mtb infection, development of the disease, or dissemination.

The role of additional T cell subpopulations during TB infection has been extensively described including Th17, Treg, Th22, and follicular T cells. Th17 cells are capable of inducing tissue inflammation and autoimmunity. Nevertheless, the precise contribution of these non-Th1/Th2 T cell subsets in TB has not been elucidated. On the other hand, cytokines related to IL-10 classically have been involved in the stimulation of Th2 responses and antibody production. However the cytokine IL-19 has an important immunoregulatory role. In this regard, the importance of IL-19, IL-27, and IL-35 in infections such as TB and in autoimmune diseases is under investigation. TNF-*α* plays a relevant role in the defense against Mtb. Several studies have established that TNF-*α* blockers have deleterious effects on the development of effective immune responses. These effects have an impact on innate and adaptive immune responses to many microorganisms, including Mtb. Nevertheless, experimental studies that have explored the effects of anti-TNF drugs in the immunopathogenesis of TB have shown variable and even controversial results.

The role of cellular and humoral immunity is important in the design of new vaccines against Mtb. However, further research focused on the mechanisms of bacterial control in individuals exposed to but not infected with Mtb and the capacity to develop latent forms of infection is needed. It is possible that the capacity to develop LTBI might be influenced by the continuous exposure to environmental mycobacteria or the continuous exposure to microbiome that is critical to induce the expression of genes associated with inflammation and antimicrobial defense. Even though the mechanisms implicated in the pathogenesis of Mtb infection are complex, a better understanding of the molecular and cellular responses against Mtb can be helpful in the development of novel therapeutic and prophylactic strategies.
